# Male Child with Van Wyk-Grumbach's Syndrome and Other Complications of Long-Standing Primary Hypothyroidism: A Case Report

**DOI:** 10.1155/2012/352751

**Published:** 2012-10-23

**Authors:** Ahmed Omran, Jing Peng, Biswas Shrestha, Muhammad Usman Ashhab, Fei Yin

**Affiliations:** ^1^Department of Pediatrics, Xiangya Hospital of Central South University, No. 87 Xiangya Road, Changsha, Hunan 410008, China; ^2^Department of Pediatrics and Neonatology, Suez Canal University, Ismailia 41522, Egypt; ^3^Department of Radiology, Third Xiangya Hospital of Central South University, Changsha, Hunan 410008, China

## Abstract

Primary hypothyroidism in the juvenile population generally leads to retardation of linear growth and delay or even arrest of puberty. However, in rare conditions, children with long-standing hypothyroidism present with signs of Van Wyk-Grumbach's syndrome (VWGS) which include juvenile hypothyroidism, delayed bone age, and pseudoprecocious puberty. We report a rare case of prepubertal male child from Asian origin, presented with long-standing untreated hypothyroidism complicated with VWGS and other complications including obesity, short stature, hepatomegaly, asymptomatic mild pericardial effusion, and pituitary hyperplasia.

## 1. Introduction

Hypothyroidism is among the most common endocrine disorders in children. In most of the hypothyroid children sexual development is delayed. However, children with severe long-standing hypothyroidism rarely present with signs of precocious puberty, the Van Wyk-Grumbach syndrome (VWGS) [1, 2]. Sexual precocity is always isosexual, characterized by breast enlargement, multicystic ovaries, and menstrual bleeding in girls and testicular enlargement with minimal penile enlargement in boys [3].

Van Wyk and Grumbach first described the combination of juvenile hypothyroidism, delayed bone age, and precocious puberty in 1960, with most of the reported cases found in girls [4–7], while very few cases reported in boys [8, 9]. This paper describes a prepubertal Asian boy with VWGS and other complications of long-standing primary hypothyroidism including obesity, short stature, hepatomegaly, mild pericardial effusion, and pituitary hyperplasia.

## 2. Case Presentation

**Figure 1:**
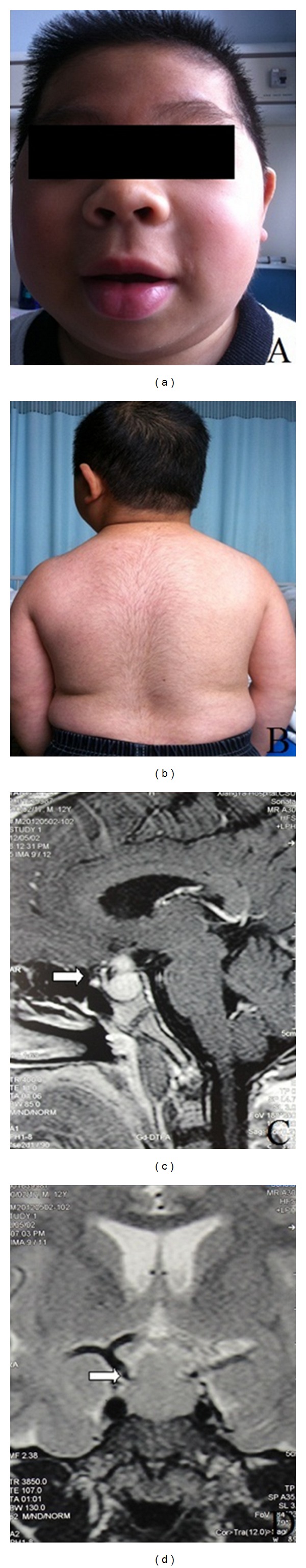
Clinical signs and MRI brain findings of the child. (a) The coarse facial features and myxedematous appearance. (b) Dark and thick hair on the back of the obese child. (c) MRI brain sagittal scan showing pituitary enlargement and homogeneous intrasellar mass with suprasellar extension. (d) MRI brain coronal scan showing the “snowman sign,” pituitary enlargement upward protruding into the suprasellar cistern, with no significant compression on optic chiasm and uniform T2 signal.

A 12-year-old Asian male child of nonconsanguineous parents was referred to the Pediatric Department of Xiangya hospital, Changsha, Hunan, China. Presented with progressive weight gain, short stature, cold intolerance, constipation, and poor school achievement with low average IQ of 76 (verbal IQ of 74 and performance IQ of 79) of 6 years duration; otherwise, clinical history was unremarkable. Physical examination during presentation revealed stable vital signs with temperature 36.8°C, heart rate 90/min, respiratory rate 20/min, and blood pressure 100/60 mm·Hg. His weight was 43 kg (>97th percentile), height 114 cm (<3rd percentile), and BMI 33.1 (>95th percentile). He had coarse facial features with myxedematous appearance (Figure 1(a)), palpable but not enlarged thyroid gland, dark and thick hair on the back (Figure 1(b)), absent axillary and pubic hair, cold extremities, and no dysmorphic features. Bilateral testicular volume was 14 ml in size as measured by Prader orchidometer and stretched penile length was 8 cm. Abdominal examination revealed hepatomegaly 5 cm below the costal margin, cardiac examination revealed slightly distant heart sound without murmurs, chest examination was normal, and fundus and visual field examinations were normal. Neurological examination was normal except the bilateral knee jerk hyperreflexia. Initial laboratory investigations are summarized in Table 1. Thyroid ultrasound (U/S) revealed multiple nodules and abdominal U/S showed hepatomegaly. While X-ray left wrist and hand revealed delayed bone age, estimated at 5 years age, chest X-ray revealed mild cardiomegaly with cardio/thoracic ratio >0.7. ECHO cardiograph showed mild pericardial effusion, with mild mitral and tricuspid regurgitation. MRI brain showed diffuse enlargement of the pituitary gland, with no limitation in the lesion size, uniform signal lesions of the pituitary gland, and consistent strength is seen with no apparent mass effect (Figures 1(c) and 1(d)). Based on physical findings, laboratory results, including high levels of TSH and ATG with low levels of T3 and T4, and radiological investigations, the diagnosis of Hashimoto's thyroiditis was made and found to be complicated with hypothyroidism, delayed bone age, and testicular enlargement. These are consistent with the diagnosis of VWGS, hepatomegaly, mild pericardial effusion, and pituitary hyperplasia. The patient was started on levothyroxine at 50 ug/day and discharged from the hospital after resolution of the pericardial effusion.

## 3. Discussion

Traditionally, long-standing primary hypothyroidism leads to both pubertal and growth delay, but in rare cases hypothyroidism leads to growth delay with paradoxically precocious puberty and delayed bone age. The syndrome consisting of primary hypothyroidism and precocious puberty was first described in 1905, but, only later in 1960, the term VWGS was coined [1].

Most of the VWGS cases reported in prepubertal girls presented with abnormal breast development, vaginal bleeding, and cystic ovaries, while it is very rare in boys and manifests with testicular enlargement [10].

Many theories exist to explain this seemingly paradoxical precocious puberty in association with primary hypothyroidism. Firstly, Van Wyk and Grumbach postulated a lack of specificity in the feedback mechanism leading to an overproduction of gonadotropins and other heterologous hormones from the pituitary hypothalamic axis including estradiol [1]. Secondly, in vitro experiments have demonstrated that TSH can weakly stimulate the FSH receptor without simultaneous stimulation of LH receptors explaining the low pre-pubertal level of LH. Therefore, high levels of TSH may act through the FSH receptor to cause gonadal stimulation and precocious sexual changes [11, 12]. Thirdly, continuous high thyrotropin-releasing hormone (TRH) concentration stimulates FSH secretion [13].

In association with our case, primary hypothyroidism in boys is associated with isosexual incomplete precocious puberty with testicular enlargement but without virilization [14]. The etiology of this unique sexual precocity remains uncertain; Jannini et al. reported direct effect of severe hypothyroidism on the pre-pubertal testis which leads to overproliferation of Sertoli cells and is responsible for the testicular enlargement in males, meanwhile the longer hypothyroidism persists, the greater is the degree of testicular damage [15].

VWGS precocious puberty has other unique features including short stature and delayed bone age which differs from other causes of precocious puberty where growth acceleration is the norm. This can be explained on the basis that thyroid hormone mediated bone maturation involves direct and indirect actions. The indirect action is mediated by the regulation of growth hormone gene expression and the insulin-like growth factor (IGF) system, while T3 directly regulates the endochondral ossification and also controls chondrocyte differentiation in the growth plate both in vitro and in vivo [16, 17].

Weight gain has been recognized as a symptom of hypothyroidism; in the absence of thyroid hormone, basal thermogenesis and resting energy expenditure are reduced by 30% to 59% [18]. Although, hypothyroidism is usually associated with loss of hair, our case showed dark and thick hair on his back which also reported in other cases with untreated primary hypothyroidism [19]. The fact that the hypertrichosis resolved after thyroxine replacement therapy establishes the causal relationship between hypertrichosis and hypothyroidism.

Pituitary enlargement secondary to hypothyroidism is a known but uncommon occurrence, with long-standing hypothyroidism; thyrotroph hyperplasia can result in the expansion of the sella turcica and the enlargement of the pituitary gland [20]. Unlike adults, children rarely have neurologic presentations secondary to sellar expansion [21]. The lack of thyroxine feedback found in uncontrolled primary hypothyroidism leads to elevated levels of TRH which causes both pituitary thyrotroph and lactotroph hypertrophy, increasing the secretion of both TSH and prolactin [22].

Elevated lipid profile, liver enzymes, and hepatomegaly in our case could be explained on the basis that hypothyroidism may cause hypercholesterolemia and play an essential role in the pathogenesis of nonalcoholic fatty liver disease (NAFLD) which represents the most common liver disease and it includes a spectrum of hepatic dysfunctions ranging from simple steatosis to non-alcoholic steatohepatitis, cirrhosis, and hepatocellular carcinoma [23]. Very recently, Chung et al. reported that both NAFLD and elevated liver enzyme levels were significantly greater in subjects with hypothyroidism compared to normal subjects [24]. The occurrence of pericardial effusion in hypothyroidism as in our case appears to be dependent on the severity of the disease. The pathogenesis of pericardial effusion in hypothyroid patients is probably due to the increased systemic capillary permeability and disturbances in electrolyte metabolism [25].

This rare syndrome represents a clinical diagnostic challenge especially in boys as long-standing primary hypothyroidism classically leads to both pubertal and growth delay. The clinical, laboratory, and radiological investigation supports the previously mentioned hypotheses that primary uncontrolled hypothyroidism can be a precursor for better understanding of this rare syndrome.

## Figures and Tables

**Table 1:** Laboratory results of the patient.

Parameter	Result	Normal values
Complete blood count	Normal	
Renal functions	Normal	
Thyroid stimulating hormone (TSH)	>100 mU/L	0.20–4.50 mU/L
Free T3	<0.4 pmol/L	3.8–8.6 pmol/L
Free T4	<5.15 pmol/L	12–22 pmol/L
Antithyroglobulin antibodies (ATG)	470 IU/mL	<60 IU/mL
Prolactin	28.2 ng/mL	2–18 ng/mL
Follicle stimulating hormone (FSH)	8.24 mIU/mL	0.3–2 mIU/mL
Luteinizing hormone (LH)	<0.05 IU/mL	0.1–6.0 IU/mL
Estradiol	19.46 pmol/L	<5 pmol/L
Serum Cortisol	15 mcg/dL	7–9 AM 6–26 mcg/dL
		4–6 PM 4–18 mcg/dL
Adrenocorticotropic hormone (ACTH)	163.4 U/L	70–300 U/L
Alanine aminotransferase (ALT)	316.5 U/L	10–40 U/L
Aspartate aminotransferase (AST)	207.5 U/L	0–45 U/L
Alkaline phosphatase (ALP)	163.4 U/L	20–140 U/L
Serum triglycerides	210 mg/dL	Up to 150 mg/dL
Total serum cholesterol	220 mg/dL	Up to 170 mg/dL
